# Association of sarcopenia with osteoporosis in Chinese patients with type 2 diabetes

**DOI:** 10.1186/s12891-024-07323-2

**Published:** 2024-03-21

**Authors:** Ke Xu, Xiaozhen Feng, Zeru Xu, Yang Pan, Ping Zhang, Hong Zhu

**Affiliations:** 1 Department of Endocrinology, The First Affiliated Hospital of Wenzhou Medical University, Wenzhou, 325000 China; 2 Department of Health Care Center, The First Affiliated Hospital of Wenzhou Medical University, Wenzhou, 325000 China; 3 Department of Endocrinology, Xining Second People’s Hospital, Xining, 810000 China

**Keywords:** Type 2 diabetes, Sarcopenia, Osteoporosis

## Abstract

**Background:**

People with type 2 diabetes mellitus (T2DM) present a higher tendency to develop sarcopenia and osteoporosis compared with the normal population. Currently, osteoporosis screening has been frequently performed among T2DM patients, but sarcopenia screening is relatively less, and the association between the two diseases remains unclear. Herein, this study aims to determine the association between sarcopenia and osteoporosis in Chinese T2DM patients.

**Methods:**

This was a retrospective study of 678 patients with T2DM in the First Affiliated Hospital of Wenzhou Medical University. The bone mineral density (BMD) and muscle mass were measured by using dual-energy X-ray absorptiometry scanning. The diagnostic criteria of sarcopenia referred to the consensus by the Asia Working Group for Sarcopenia (AWGS).

**Result:**

Among T2DM patients, the proportion of the sarcopenia population complicated with osteoporosis was higher than that of the non-sarcopenia (30.9% vs. 8.6% in men and 46.9% vs. 33.9% in women), but only significantly in men. The BMD of the hip and femoral neck was positively correlated with skeletal muscle mass index (SMI), grip strength, and gait speed (*P* < 0.01). After adjusting all covariates, the association between sarcopenia and BMD showed odds ratios of 0.43 (95% CI:0.28–0.66) for the femoral neck and 0.49 (95% CI:0.32–0.73) for the hip.

**Conclusions:**

The BMD of the hip and femoral neck in T2DM patients is related to sarcopenia-related indicators and represents an independent protective factor for sarcopenia. To reduce the risk of falls, fractures, and weakness, it is necessary to take sarcopenia assessment in people with T2DM and osteopenia/osteoporosis.

## Introduction

Both muscle and bone belong to the motor system, which originate from the mesoderm and share the same interstitial precursor. Muscle and bone not only have an adjacent anatomical position, but also are under the regulation of common genes, common paracrine and endocrine mechanisms, and similar molecular signaling pathways [1, 2]. Exploring the association between muscle and bone and thereby performing certain interventions and treatments for sarcopenia and osteopenia can effectively reduce the rate of disability and mortality and improve the quality of life of the elderly.

Osteoporosis and sarcopenia are two chronic musculoskeletal disorders frequently occurring in the elderly. With global demographics trending towards an aging population, the prevalence of osteoporosis and sarcopenia is on the rise year by year, carrying significant risks of falls, fractures, and hospitalization in the elderly. Sarcopenia is defined by progressive systemic muscle mass reduction and/or muscle strength decline or muscle physiological function decline with aging [3]. Osteoporosis is a systemic metabolic bone disease characterized by bone mass reduction and bone tissue microstructure destruction, leading to bone fragility and fracture susceptibility [4]. Muscle and bone mass loss is a common pathological condition in the elderly.

Epidemiological data indicate that diabetes mellitus can exert an adverse impact on bones, known as diabetes mellitus-induced osteoporosis, which represents that osteoporosis is comprehensively affected by the additional fracture burden caused by diabetes mellitus. Diabetes mellitus, both type 1 and type 2 (T2DM), is associated with an increased risk of fracture, and some anti-diabetic drugs may also increase the fracture risk [5]. The skeletal muscle is the largest and most energy-consuming organ in the human body, which plays a vital role in energy metabolism and glucose intake. The prevalence of sarcopenia in patients with diabetes mellitus reaches 7-29.3% [6]. Currently, osteoporosis screening has been carried out widely in diabetes patients. Nevertheless, the presence of sarcopenia related to diabetes is not well acknowledged and the association between the two diseases is not clear. Hence, this study aims to determine the association between sarcopenia and osteoporosis in T2DM patients.

## Materials and methods

### Research subjects

A total of 734 T2DM patients hospitalized in the Department of Endocrinology of the First Affiliated Hospital of Wenzhou Medical University from June 2020 to June 2021 were selected, and 678 patients with complete information were finally included in this study for analysis. Inclusion criteria: (1) meet the diagnostic criteria for T2DM issued by World Health Organization in 1999; (2) age ≥ 50 years old; (3) cooperate to receive the evaluation of muscle mass, muscle strength, and physical functioning. Exclusion criteria: (1) age < 50 years old; (2) T1DM, gestational diabetes, or other special types of diabetes; (3) malignant tumors; (4) autoimmune diseases; (5) severe heart, liver, or kidney diseases; (6) medications that may affect body composition, such as diuretics, adrenocortical hormones, etc.; (7) edema of both lower limbs; (8) stay in bed for a long time. This study was approved by the Ethics Committee of the Hospital and informed consent was obtained from all subjects who met the inclusion criteria.

### Assessment of muscle mass, muscle strength, and physical functioning

#### Muscle mass

Muscle mass was assessed using the dual-energy x-ray absorptiometry (DXA) (Prodigy Primo-81013GA series, GE Company, USA) operated by an experienced radiologist. The patient was instructed to lie flat on the console in a hospital gown, without carrying any metal products. Appendicular skeletal mass (ASM; kg) was automatically uploaded by the system, namely, the sum of the lean soft tissue (LST) from the upper and lower limb [7]. Then the ASM was further processed to obtain the skeletal muscle mass index (SMI) and ASM/BMI, SMI = ASM/Height^2^.

#### Muscle strength

Muscle strength was evaluated by a specially assigned person using an electronic grip strength tester (TH-01, Xiangshan Company, China), with an accuracy of 0.1 kg. The patient took a sitting position, kept his arms on his side naturally, and flexed his elbows about 90 degrees. The patient was instructed to hold the electronic grip tester with the dominant hand and squeeze the handle as hard as possible. The grip strength test was conducted 3 times for each patient, with an interval of about 1 min. All measurement values were recorded, with the maximum value determined as the muscle strength.

#### Physical functioning

Physical functioning was evaluated using the 6 m walking test by a specially assigned person. The patient walked 6 m at a normal gait speed. If necessary, the patient was allowed to use a walking stick or walker aid, and could not accelerate or decelerate on the way. The walking test was carried out twice, accurate to 0.1 m/s. All measurement values were recorded, and the average value was taken to indicate physical functioning.

### Clinical data collection

All patient data stored in the hospital’s electronic medical record system during hospitalization were collected. General clinical data included demographic information, current medical history, past medical history, medication, etc. Anthropometric data were collected by special personnel. Height (cm) and weight (kg) were accurate to 0.1 cm and 0.1 kg respectively, and body mass index (BMI) = weight/height^2^. Waist circumference measurement: take the midpoint of the line between the lowest edge of the rib arch and the highest point of the iliac bone, horizontally surround the waist for 1 week, and measure the circumference at the end of calm exhalation. The measurement value of waist circumference was accurate to 0.1 cm. BMD was measured using the DXA (Prodigy Advance, GE Company, USA) operated by an experienced radiologist. For the laboratory examination, after fasting overnight, blood samples were taken from the antecubital vein and centrifuged at 5000 rpm for 20 min. Before conducting the assay, the plasma was stored in freezing tubes at − 80°C., The laboratory examination was completed in the Test Center of the First Affiliated Hospital of Wenzhou Medical University. Laboratory indicators in this study included glycated hemoglobin (HbA1c), 25-hydroxyvitamin D (25(OH)D), estradiol, testosterone, and insulin-like growth factor-1 (IGF-1).

### Diagnostic criteria

The diagnosis of sarcopenia adopted the diagnostic algorithm of the Asia Working Group for Sarcopenia (AWGS) [8]. Sarcopenia was defined as muscle mass reduction combined with muscle strength decline or physical functioning impairment, that is, male: SMI < 7.0 kg/m^2^, grip strength < 28 kg; female: SMI < 5.4 kg/m, grip strength < 18 kg; gait speed < 1 m/s.

### Statistical methods

Statistical analysis was performed using SPSS (IBM SPSS Statistics for Windows, version. 22.0. Armonk, NY: IBM Corp). Data were presented as mean ± standard deviation (X ± S) and percentage. The differences between groups were compared using the t-test, and categorical data, using χ2 test. Pearson’s correlation coefficient was used to analyze the correlation between BMD and sarcopenia-related indicators. Binary logistic regression was used to comprehensively analyze the risk factors of sarcopenia. A value of *P* < 0.05 was indicative of statistical significance.

## Results

In our study, a total of 678 T2DM patients were enrolled, including 447 males, aged 56.99 ± 13.22 years old, and 231 females, aged 62.07 ± 11.66 years old. Table 1 shows the characteristics of men and women affected by sarcopenia. Men with sarcopenia were older, higher estradiol, higher rate of stroke and coronary heart disease, and had lower HC, ASM, SMI. In women, the sarcopenia group had lower BMI, WC, HC, ASM, SMI, Grip, Gait speed and BMD of femoral neck, Hip. No difference was found in the prevalence of sarcopenia according to smoking, drinking status and HbA1C both in men and women. The proportion of the sarcopenia population complicated with osteoporosis was higher than that of the non-sarcopenia(30.9% vs. 8.6% in men and 46.9% vs. 33.9% in women), but only significantly in men. Table 2 shows the correlation between BMD at different sites and sarcopenia-related indicators. The BMD of L1-L4 was significantly positively correlated with ASM, SMI, and grip strength, but had no significant correlation with gait speed. The BMD of the femoral neck and hip were positively correlated with SMI, grip strength, and gait speed (*P* < 0.01).

**Table 1:** Clinical characteristics of study participants

Variables	Men(*n* = 447)			Women(*n* = 231)		
	Sarcopenia (*n* = 84)	Non-sarcopenia (*n* = 363)	p	Sarcopenia (*n* = 34)	Non-sarcopenia (*n* = 197)	p
Age(y)	63.71 ± 14.51	54.53 ± 12.77	0.03	64.59 ± 10.69	61.31 ± 11.79	0.12
Education level(%)			0.39			0.17
Junior high school or below	76.2%(64)	71.6%(260)		97.1%(33)	89.8%(177)	
High school and above	23.8%(20)	28.4%(103)		2.9%(1)	10.2%(20)	
Drinking (%)	52.4%(44)	55.6%(202)	0.58	2.9%(1)	5.1%(10)	0.58
Smoking (%)	67.9%(57)	58.4%(212)	0.11	2.9%(1)	0.5%(1)	0.15
Stroke (%)	20.2%(17)	7.7%(28)	< 0.01	11.8%(4)	12.2%(24)	0.94
Coronary Heart Disease (%)	19.0%(16)	7.2%(26)	< 0.01	2.9%(1)	7.6%(15)	0.32
BMD(SD)						
L1-L4	-0.71 ± 1.76	-0.33 ± 1.43	0.18	-1.88 ± 1.36	-1.34 ± 1.54	0.05
femoral neck	-1.67 ± 1.08	-0.79 ± 1.11	0.95	-2.02 ± 1.06	-1.36 ± 1.15	< 0.01
Hip	-1.31 ± 1.05	-0.57 ± 1.03	0.93	-1.73 ± 1.19	-0.87 ± 1.26	< 0.01
BMI	21.38 ± 2.83	24.33 ± 3.26	0.20	20.45 ± 2.89	23.72 ± 3.13	< 0.01
WC (cm)	85.96 ± 9.79	91.66 ± 8.99	0.32	84.10 ± 8.34	90.16 ± 9.33	< 0.01
HC (cm)	90.00 ± 7.93	95.22 ± 6.16	< 0.01	90.24 ± 5.73	94.19 ± 7.09	< 0.01
SBP (mmHg)	142.28 ± 21.96	131.29 ± 18.72	0.05	141.44 ± 22.76	136.56 ± 19.69	0.24
DBP(mmHg)	78.53 ± 12.31	76.91 ± 11.29	0.22	77.33 ± 10.06	75.69 ± 9.47	0.40
HbA1c(%)	9.49 ± 2.55	9.74 ± 2.39	0.99	9.29 ± 21.50	9.27 ± 2.20	0.96
25(OH)D (nmol/L)	49.89 ± 20.93	59.42 ± 21.19	0.94	51.92 ± 19.70	56.19 ± 19.35	0.25
Estradiol(pmol/L)	201.39 ± 89.96	170.43 ± 66.34	< 0.01	143.18 ± 148.75	141.92 ± 127.77	0.96
Testosterone(nmol/L)	10.14 ± 4.95	10.98 ± 4.34	0.35	0.90 ± 0.59	1.01 ± 0.97	0.54
IGF-1 (ng/mL)	165.78 ± 116.71	163.36 ± 55.84	0.01	153.90 ± 51.01	129.42 ± 56.57	0.21
ASM (kg)	17.59 ± 1.98	23.21 ± 3.07	< 0.01	12.15 ± 1.29	16.51 ± 2.18	< 0.01
SMI (kg/m2)	6.36 ± 0.55	8.09 ± 0.94	< 0.01	4.93 ± 0.41	6.67 ± 0.77	< 0.01
Grip(kg)	22.62 ± 7.41	36.06 ± 7.98	0.32	14.40 ± 5.14	20.60 ± 5.45	< 0.01
Gait speed(m/s)	0.81 ± 0.24	1.08 ± 0.20	0.12	0.82 ± 0.17	0.92 ± 0.19	0.02
Osteopenia (%)	39.5%(32)	32.2%(112)	0.20	37.5%(12)	36.5%(69)	0.91
Osteoporosis (%)	30.9%(25)	8.6%(30)	< 0.01	46.9%(15)	33.9%(64)	0.15

SBP, systolic blood pressure; DBP, diastolic blood pressure; WC, waist circumference; BMI, body mass index; WC, waist circumference; HC, hip circumference; BMD, bone mineral density; ASM, appendicular skeletal muscle mass; SMI, skeletal muscle mass index; HbA1c, glycosylated hemoglobin; 25(OH)D, 25-hydroxyvitamin D; IGF-1, insulin-like growth factor-1; The differences between groups were compared using the t-test, and categorical data, using χ2 test. Data were presented as means ± SD or number (%)

**Table 2:** Correlation between bone mineral density in different parts and sarcopenia-related indicators

BMD	ASM (kg)	SMI (kg/m2)	Grip(kg)	Gait speed(m/s)
L1-L4	0.31**	0.25**	0.24**	**0.08**
femoral neck	0.37**	0.31**	0.38**	0.21**
Hip	0.31**	0.30**	0.29**	0.16**

BMD, bone mineral density; ASM, appendicular skeletal muscle mass; SMI, skeletal muscle mass index. Statistic performed by pearson’s correlation, **p < 0.01

Table 3 shows the correlation between BMD at different sites and sarcopenia. In models 1(crude), 2(adjusted for age and sex), and 3(adjusted for variables in model 2 + education level, smoking status, drinking status, stroke, coronary heart disease), the BMD of L1-L4, hip, and femoral neck was a protective factor for sarcopenia. After adjusting all covariates (adjusted for variables in model 3 + Hba1c, 25(OH)D, IGF-1, Estradiol, Testosterone), there was no significant correlation between the BMD of L1-L4 and sarcopenia (*P* = 0.05). The correlation between sarcopenia and BMD showed odds ratios of 0.43 (95% CI:0.28–0.66) for the femoral neck and 0.49 (95% CI:0.32–0.73) for the hip.

**Table 3:** Correlation between BMD at different sites and sarcopenia

OR(95%CI)	BMD		
	L1-L4	Femoral neck	Hip
Model 1	0.86(0.75–0.99)	0.53(0.43–0.65)	0.54(0.45–0.66)
P-value	0.03	< 0.01	< 0.01
Model 2	0.84(0.73–0.97)	0.56(0.45–0.69)	0.57(0.46–0.71)
P-value	0.02	< 0.01	< 0.01
Model 3	0.87(0.76–0.99)	0.57(0.46–0.71)	0.58(0.47–0.72)
P-value	0.04	< 0.01	< 0.01
Model 4	0.75(0.57-1.00)	0.43(0.28–0.66)	0.49(0.32–0.73)
P-value	0.05	< 0.01	< 0.01

Model 1: crude; Model 2: adjusted for age and sex; Model 3: adjusted for variables in model 2 + education level, smoking status, drinking status, stroke, coronary Heart Disease; Model 4: adjusted for variables in model 3 + Hba1c, 25(OH)D, IGF-1, Estradiol, Testosterone. Statistic performed by binary logistic regression

## Discussion

This study mainly explored the association between BMD at different sites and sarcopenia in patients with T2DM. The results demonstrated that the proportion of patients with sarcopenia who suffered from osteopenia or osteoporosis at the same time was higher than that of patients without sarcopenia, especially in men. The BMD of the hip and femoral neck was positively correlated with sarcopenia-related indicators and represented an independent protective factor for sarcopenia.

Sarcopenia is an age-related condition, accompanied by a progressive decline in muscle mass and loss of muscle function, followed by physical strength reduction, quality of life decline, and risk of falls. T2DM is a chronic metabolic disease, and elderly diabetics are highly susceptible to sarcopenia. In addition to microvascular and macrovascular complications, sarcopenia has been described as another notable complication of diabetes in the elderly. Compared with non-diabetic patients, T2DM patients present significantly decreased muscle mass and muscle strength with aging [9]. A meta-analysis has shown that the prevalence of sarcopenia in T2DM patients is 18% (95% CI 0.15–0.22) [10], similar to that obtained by our study (17.40%). The high prevalence of sarcopenia in T2DM patients may be explained by different mechanisms. Since the insulin sensitivity related to sarcopenia is impaired, the anabolism of insulin in skeletal muscle may be gradually lost in T2DM patients. In addition, the impairment of insulin effect may lead to the reduction of protein synthesis and the increase of protein degradation, resulting in the reduction of muscle mass and strength [11]. Chronic hyperglycemia promotes the accumulation of advanced glycosylation end products (AGEs) in skeletal muscle, and AGEs are related to the reduction of grip strength, leg extension strength, and gait speed [12]. Also, the pathogenesis of diabetes can be attributed to the increase of inflammatory cytokines, which act on muscles and lead to the reduction of muscle strength and function [13]. IGF-1, estradiol and testosterone are important endocrine factors that affect muscle and bone growth and development at the same time. Chen et al. [14] studied 1839 residents older than 50 years old and showed that the level of serum IGF-1 was positively correlated with muscle mass and hand grip strength. At the same time, the higher the level of serum IGF-1, the greater the bone mineral density of spine and femoral neck. Estrogen maintains the dynamic balance of bone formation and bone resorption, and can affect the differentiation of bone marrow mesenchymal stem cells into osteoblasts; The sharp decline of estrogen levels in menopausal women will accelerate the age-dependent degradation of bone. The lack of testosterone leads to the decline of body mass, muscle mass and muscle strength; Testosterone can also regulate the metabolic function of skeletal muscle and the repair of skeletal muscle injury [15].

Despite the high prevalence of sarcopenia in T2DM patients, only a few studies have revealed the correlation between muscle loss and blood glucose control [16]. Consistent with the findings of most studies, our results found that the prevalence of sarcopenia was not correlated with HbA1C levels. It may be due to the fact that HbA1c levels cannot fully reflect all aspects of daily blood glucose status, especially hypoglycemic attack and daily blood glucose fluctuation, which may be risk factors for sarcopenia [13]. Meanwhile, diabetes is also considered an independent risk factor for fragility fractures [17]. The increase in blood glucose leads to calcium homeostasis imbalance by inhibiting bone formation and accelerating bone absorption, manifested as primary and secondary spongy trabecular bone loss clinically. High glucose has been confirmed to enhance osteoblast apoptosis. Hyperglycemia may inhibit genes related to osteogenic transdifferentiation and bone resorption, including metalloproteinase (MMP) 9 and carbonic anhydrase II (CAII), and also decrease the expressions of osteogenesis-related genes such as Runx2 and alkaline phosphatase (ALP) [18]. When mesenchymal progenitor cells are exposed to high glucose, the Notch2 signaling pathway is activated to inhibit osteogenesis [19]. In addition, the accumulation of AGEs may lead to bone matrix damage, bone strength damage, and low bone transformation rate in diabetics [20], while the accumulation of AGEs is also an important contributor to inflammatory events leading to diabetes and its complications, which may also be one of the mechanisms inducing osteoporosis in diabetics.

Sarcopenia and osteoporosis are two pathological conditions that seriously compromise the health and quality of life of the elderly. Both muscle and bone originate from mesenchymal stem cells, and the developmental homology makes them closely related. Muscle and bone are not only adjacent to each other in anatomical position but also regulated by common genes, with common paracrine and endocrine mechanisms and similar molecular signaling pathways [2]. Binkley et al. [19] put forward the concept of osteosarcopenia considering that sarcopenia and osteoporosis share the same pathophysiological basis and exert the same adverse effects on the physiological health of the elderly. In our study, the prevalence of complicated osteopenia was 38.9% in T2DM patients with sarcopenia, and the prevalence of complicated osteoporosis was 35.4%, both significantly higher than that in non-sarcopenia patients. In addition, when participants stratified by sex, the proportion of the sarcopenia population complicated with osteoporosis (30.9% vs. 8.6%) was only significantly higher than that of the non-sarcopenia in men. In addition, we also found that the BMD of L1-L4 was positively correlated with ASM and SMI. The BMD of the femoral neck and hip was positively correlated with SMI, grip strength, and gait speed. Previous studies have also unveiled a link between muscle and bone [21, 22]. However, these studies mostly focus on the protection of muscle to bone but ignore the role of bone to muscle. In our study, we first time found that the BMD of hip (0.49,95% CI:0.32–0.73), and femoral neck (0.43,95% CI:0.28–0.66) was an independent protective factor for sarcopenia. Patients with low BMD hip or femoral neck should also pay more attention to their muscle condition. With the in-depth exploration of the musculoskeletal system, researchers have gradually realized that both bone and muscle tissues have secretory functions. Bone and muscle can secrete a variety of biochemical factors, named myokines and osteokines respectively, which perform an important role in the interaction between muscle and bone. The cells in bone tissues can secrete multiple osteokines including osteocalcin (OCN), prostaglandin E2 (PGE2), fibroblast growth factor-23 (FGF23), receptor activator of nuclear factor-κB ligand (RANKL), and sclerostin (SOST). OCN is synthesized and secreted by osteoblasts and binds to G protein coupled receptor C family 6 group A (GPRC6A). OCN increases muscle mass and regulates muscle function via the GPRC6A/AMPK/mTOR/S6 kinase pathway. GPRC6A gene knockout mice have decreased muscle mass, while the muscle mass is increased in embryonic stem phosphatase (Esp) gene knockout mice, indicating the promoting effect of OCN on muscle mass and muscle function [23]. FGF23 is an endocrine factor secreted by osteoblasts and osteocytes. PGE2 secreted by osteocytes is 1000 times higher than that secreted by myocytes. The excessive PGE2 in bone cells interacts with damaged muscles, which facilitates muscle regeneration and repair [24]. The high level of FGF23 in serum negatively regulates the synthesis of 1,25-dihydroxyvitamin D [1,25 (OH) D] [25], triggering cardiovascular diseases, weakness, disability, and other adverse consequences. A study on the elderly has shown that the high level of FGF23 in serum is independently related to the debilitating condition and death risk of the elderly [26], but the regulatory role of FGF23 on skeletal muscle needs further exploration.

In addition to the paracrine interaction between muscle and bone, they also have common signaling pathway regulation. The NF-κB pathway is one of the most important signaling pathways in muscle, and its activation leads to skeletal muscle atrophy. Some scholars believe that NF-κB activation-induced muscle atrophy may be related to the increase of inflammatory factors such as TNF-α and IL-6, and blocking this pathway may represent a promising target to treat muscle atrophy. In bone tissue, the NF-κB signaling pathway participates in corticosteroid-induced osteocyte apoptosis and mediates RANKL-induced osteoclast formation, differentiation, and maturation. Moreover, the Wnt/β-catenin signaling pathway is deemed as one of the signaling pathways that simultaneously regulates bone and muscle metabolism. Wnt signal can regulate osteoblast activity, muscle formation, and regeneration in the embryonic stage [27]. These systemic and local factors directly or indirectly affect the musculoskeletal unit, forming osteosarcopenia, which significantly increases the risk of falls and fractures. Therefore, early and effective prevention and treatment are especially crucial for T2DM patients.

This study has several limitations. Firstly, as a cross-sectional study, this study failed to determine the causal relationship between sarcopenia and osteoporosis. Secondly, although we adjusted smoking, alcohol, education, and other factors in the statistical analysis, some important questionnaire contents were still not considered, such as family economic level, dietary habits, exercise frequency, sunshine time, etc. Thirdly, our study subjects were limited to the Chinese population, and the conclusions may have certain disparities among people of different races and latitudes.

## Research conclusions

This study demonstrated that T2DM patients in China complicated with sarcopenia had a higher tendency to suffer from osteoporosis compared with those without sarcopenia, but only significantly in men. Men with sarcopenia were older, higher estradiol, higher rate of stroke and coronary heart disease, and had lower HC, ASM, SMI. In women, the sarcopenia group had lower BMI, WC, HC, ASM, SMI, Grip, Gait speed and BMD of femoral neck, Hip. The BMD of the hip and femoral neck was positively correlated with sarcopenia-related indicators and represented an independent protective factor for sarcopenia. To reduce the risk of falls, fractures, and weakness in these T2DM patients, it is necessary to take sarcopenia assessment in people with T2DM and osteopenia/osteoporosis.

## Data Availability

The datasets generated and/or analysed during the current study are not publicly available but are available from the corresponding author on reasonable request.
